# Supplementary material for: Molecular and clinical profiling in a large cohort of Asian Indians with glycogen storage disorders
Source: PLoS One. 2022 Jul 14;17(7):e0270373. doi: 10.1371/journal.pone.0270373 (PMC9282608; doi:10.1371/journal.pone.0270373)
Supplement: S1 File — (DOCX) [file pone.0270373.s001.docx]

| Exon | Primer 5’-3’ | Tm |
| --- | --- | --- |
| *G6PC EX 1F* | TAGCAGAGCAATCACCA | 58 |
| *G6PC EX 1R* | ATCAGAAGTTGCTTTCCCCA |  |
| *G6PC EX 2F* | TGTGAAATCCTTCTCAGGCTA | 58 |
| *G6PC EX 2R* | TTGAGGGGACATGAGGAGAG |  |
| *G6PC EX 3F* | ATGGGTGGATGGATG | 60 |
| *G6PC EX 3R* | ATTCTTCCTCACATCCCCCT |  |
| *G6PC EX 4F* | TAAGTTTGCCAGGCTCCAAC | 62 |
| *G6PC EX 4R* | AAAATCCCAGCATTCCAGC |  |
| *G6PC EX 5aF* | CCAAACCCACCTCTAGCAAA | 62 |
| *G6PC EX 5a R* | CCATTCCGCCTCAGCTCT |  |
| *G6PC EX 5bF* | CATTGACACCACACCCTTT | 62 |
| *G6PC EX 5bR* | TTTGAAGCAATGGGCACTG |  |

SUPPLEMENTARY FILE

Table 1 : GSD 1a primer details

| Exon | Primer 5’-3’ | Tm |
| --- | --- | --- |
| *SLC37A4* EX 1F | GTGGTCAGAGGCTGTGCGT | 60 |
| *SLC37A4* EX 1R | TGGGGACACAGAACCAGCCAG |  |
| *SLC37A4* EX 2F | TCATTGCTCCTGTGTTTCTCC | 56 |
| *SLC37A4* EX 2R | GATTGTCATAGAGGATGCCTA |  |
| *SLC37A4* EX 3F | CATCTGACCCCACCCTCAACAT | 60 |
| *SLC37A4* EX 3R | GATGTTTGTTGAAGACTAGGCG |  |
| *SLC37A4* EX 4F | AGCAGTCAGGGCAGAGCCTGA | 58 |
| *SLC37A4* EX 4R | AAGGAGCAGGAACCAATGCAG |  |
| *SLC37A4* EX 5F | CCAATGTGTAACACCCTCCCA | 57 |
| *SLC37A4* EX 5R | CTTCACGGCGTGGCTGGGACA |  |
| *SLC37A4* EX 6F | TGTTCTGAGGACGTGACATTG | 60 |
| *SLC37A4* EX 6R | AAGGGCCACTGACTCTGTCTC |  |
| *SLC37A4* EX 8F | CAGGTCGGCTTTCCGACTCTG | 57 |
| *SLC37A4* EX 8R | TTGCATATAACCAGTGAGAA |  |
| *SLC37A4* EX 9F | ACTGGCTTAGGTTCTTCCCTT | 54 |
| *SLC37A4* EX 9R | GCACCATCCCACGGTGGCCTT |  |

Table 2 : GSD 1b primer details

| Exon | Primer 5’-3’ | Tm |
| --- | --- | --- |
| *AGL* EX 3F | CGAACATGTAAGTGCCGCTGTCA | 58.4 |
| *AGL* EX 3R | AGAACACAGCACCATCTTTGCACAA |  |
| *AGL* EX 4F | GTAGTGCCAAAACAGCATTAGGTTTGC | 58 |
| *AGL* EX 4R | GCACTGCCATGGTTCATACAGTAACAT |  |
| *AGL* EX 5F | TTCCATTAAGTTTTGTTGCAAC | 58 |
| *AGL* EX 5R | CTGCAATGAGAGAATGGACTAATACAC |  |
| *AGL* EX 6F | TGAACCCAAGTGTTTGACCTCTTTTCC | 55.7 |
| *AGL* EX 6R | CCTTTCTCTTATTTGTGTGTATATGTG |  |
| *AGL* EX 7F | AACTTTTCCTGTAACAGTATCATCG | 50 |
| *AGL* EX 7R | AATACAGGTTCTAAGTAATTTTCAACC |  |
| *AGL* EX 8F | GCACTTTGGCGTTTCTCCTGTGA | 66 |
| *AGL* EX 8R | GACGTTACCCAAAAGAGAGTTTTCCCT |  |
| *AGL* EX 9F | GGGAGGAGGTAGGAGGATAC | 58 |
| *AGL* EX 9R | CACATATAGAAACATGGCCCACACACA |  |
| *AGL* EX 10F | CTGTGTGTGGGCCATGTTTCTATATGT | 65 |
| *AGL* EX 10R | TTCCCAAAAGGCAATTAACTGCCTGAA |  |
| *AGL* EX 11F | CTGCATTTCTCCATCTGCTCTAGCAA | 63 |
| *AGL* EX 11R | ATTTAAGAAATGTACTGAACTCACATG |  |
| *AGL* EX 12F | CATCCTGCTAGATTTACTCAAAAAGCC | 65 |
| *AGL* EX 12R | ACCAATAGACTAATGGGGAAGAAAATC |  |
| *AGL* EX 13F | TTAAAAACCAGTGTTTCCTTGAAG | 58 |
| *AGL* EX 13R | AATGCTTGTGTCCAACTAGC |  |
| *AGL* EX 14F | TATGTCAAATCATGCCTCCTTTTGTC | 63 |
| *AGL* EX 14R | GAAATGAGGTATCTTACCCCAAAGTAG |  |
| *AGL* EX 15F | CCATTTCTCCAGTTAAGTTATGGG | 63 |
| *AGL* EX 15R | TGGGTATGATTGTGACCAAGTGTCAGA |  |
| *AGL* EX 16F | GGTCACAATCATACCCATATACTTC | 62 |
| *AGL* EX 16R | AAACCACTGAAATCTGGACAAAGG |  |
| *AGL* EX 17F | CTATGGCATGTTGTGCTAGTGGAAGT | 63 |
| *AGL* EX 17R | TCCACATACACCTGAGAAGCAGAAAGA |  |
| *AGL* EX 18F | AGGAGCTTGGAGCCAAGGGTTT | 65 |
| *AGL* EX 18R | CCATCATACCTGGCCAAGTTACCAAA |  |
| *AGL* EX 19F | GATTTGAAACCACTTTAGCCTTCC | 65 |
| *AGL* EX 19R | TGTGGCAACTCCAGCTTGTTTAAC |  |
| *AGL* EX 20F | TGGGACTCTCATCTTACTACTGTG | 63 |
| *AGL* EX 20R | GCATGTGGATCAAGACTAACTCTG |  |
| *AGL* EX 21F | TTGAAAACTTGTCTCCAGGAAGTG | 63 |
| *AGL* EX 21R | TGGACCGTACTTTGAGTAGCAAGGAT |  |
| *AGL* EX 22F | GAATGCTGAGTTCCTAAAACATACAC | 63 |
| *AGL* EX 22R | TGCAACCCAAGTAGGCATACTCTGA |  |
| *AGL* EX 23F | TTGTGGACTGGGTAGCCCTTGT | 65 |
| *AGL* EX 23R | GAAGGAAGGAGGAAAATGGTTCAGGTT |  |
| *AGL* EX 24F | CCTCCTTCCTTCATCATCTTTCAG | 63 |
| *AGL* EX 24R | CTATCCACCTACAAGCCTTTTCAG |  |
| *AGL* EX 25F | CTATCCACCTACAAGCCTTTTCAG | 54 |
| *AGL* EX 25R | AAAATCTTGAGTAGCATTACAAGC |  |
| *AGL* EX 26F | TGGGTGAAATGAAAGCAGTTTTG | 62 |
| *AGL* EX 26R | AAAATCTTGAGTAGCATTACAAGC |  |
| *AGL* EX 27F | ACCCCAGGTTTAGAGTAACTGTTC | 62 |
| *AGL* EX 27R | GGTGCCAAATCAATACTGACATTTG |  |
| *AGL* EX 28F | CTACCTAAAGAAAATACAGCTCCC | 65 |
| *AGL* EX 28R | ATTATATCGTGAGGTTTGGCACAC |  |
| *AGL* EX 29F | CAAAAGTGACTGGTTTTTGTCTTC | 65 |
| *AGL* EX 29R | AGATGAAGGGAAGAAGGCAGGGAAAT |  |
| *AGL* EX 30F | TTCATTACAATTGTTTACCGAATGCCC | 62 |
| *AGL* EX 30R | GGGTTTTCCGATATTAGCTGATAG |  |
| *AGL* EX 31F | CTGGCCTCACCCCAATTCCTATTTC | 63 |
| *AGL* EX 31R | AACAAATGGGAATAAGGAACTAAGC |  |
| *AGL* EX 32F | ATTATATCGTGAGGTTTGGCACAC | 67 |
| *AGL* EX 32R | AGATGGCATCTCCTTTTGTTGCCC |  |
| *AGL* EX 33F | CAAACTGAGCTTTAGAGTGGTTGTCCT | 63 |
| *AGL* EX 33R | AGGCCACAGCCACTCCTAAAAAAG |  |
| *AGL* EX 34F | AGATGAAGGGAAGAAGGCAGGGAAAT | 62 |
| *AGL* EX 34R | CCTAGGGCATACAGAAATCAATTC |  |
| *AGL* EX 35F | TTCATTACAATTGTTTACCGAATGCCC | 65 |
| *AGL* EX 35R | AACTTGAGCCTGTGCATATAAGGCATT |  |

Table 3 : GSD III primer details

| Exon | Primer 5’-3’ | Tm |
| --- | --- | --- |
| *PYGL* EX4F | ATTGTGCAGTAGTATCTGTGC | **56** |
| *PYGL* EX4R | TAGAACTCACTGATACCAACC |  |
| *PYGL* EX7F | TGTGTTGAAATCCTTTGG | **55** |
| *PYGL* EX7R | AATACGGAGCTTGTTCTGC |  |
| *PYGL* EX13F ext | CCCATGTTCTGTGTGATACG | **61** |
| *PYGL* EX13R ext | GTTGCAGTGAGCCGAGATCG |  |
| *PYGL* EX13f int | TACACTGTGGTAGGTGACAG | **61** |
| *PYGL* EX13R int | CACTCCAGCCTGGGCAACAG |  |

Table 4 : GSD VI primer details

| Exon | Primer 5’-3’ | Tm |
| --- | --- | --- |
| PHKA2 EX 2F | GGCATGTGATGTCTCACCC | 59 |
| PHKA2 EX 2R | AATAGAGGAGAGGCCTACACC |  |
| PHKA2 EX26F | ACGTCCACCTGACCCTACC | 63 |
| PHKA2 EX26R | ATGCCCCACAGTGCTGGTTC |  |

Table 5 : GSD Xia primer details

| Exon | Primer 5’-3’ | Tm |
| --- | --- | --- |
| PHKB EX1F | ATTGCTGACAGGCGGCC | 60 |
| PHKB EX1R | ACTCGGGGCACAGGTTCAT |  |
| PHKB EX6F | CACTATTAGAGCAAATGACT | 51 |
| PHKB EX6R | GCAATGCATTGAAGATATAA |  |
| PHKB EX12F | AAATAATTGTAACTGGGCTA | 50 |
| PHKB EX12R | ATTTCATATCAGGGATCATT |  |
| PHKB EX14F | ATGGCCTAGTATGTGGTATATC | 55 |
| PHKB EX14R | GGAGAAACCTTTCCGTCAG |  |

Table 6: GSD IXb primer details

| Exon | Primer 5’-3’ | Tm |
| --- | --- | --- |
| PHKG2 EX3F | TCATTCCACTAAAGAGTG | 50 |
| PHKG2 EX3R | TGAGCAGGATGGGTCCAG |  |
| PHKG2 EX6F | AGGAAACCAGGTAAGGGTTGAGC | 62 |
| PHKG2 EX6R | TTGGTACTCAGACCCAGCCAAC |  |
| PHKG2 EX7F | AGATGGGAACACTGGTAGTC | 58 |
| PHKG2 EX7R | AAGAGACTGGAGTCAGGCG |  |

Table 7: GSD IXc primer details

| Patient | Age / sex | Consanguinity | GSD type | Gene | Mutation | ACMG Significance | Sift | Provean | Polyphen |  |
| --- | --- | --- | --- | --- | --- | --- | --- | --- | --- | --- |
| P 01 | 3 y/ F | YES | Ia | *G6PC1* | p.H119D | Pathogenic | 0.002 | -8.1 | 0.937 |  |
| P 02 | 14y/M | YES | Ia | *G6PC1* | p.G222R | Pathogenic | 0.001 | -4.8 | 1 |  |
| P 03 | 5m/M | YES | Ia | *G6PC1* | p.K76M | Likely pathogenic | 0 | -5.43 | 0.012 |  |
| P 04 | 1 m/ M | YES | Ia | *G6PC1* | p.W156X | Pathogenic | NA | NA | 0.948 |  |
|  |  |  |  |  |  |  | NA | NA | NA |  |
| P 05 | 1 y/ F | YES | Ia | *G6PC1* | P.G184X | Pathogenic | NA | NA | NA |  |
| P 06 | 10m/F | na | Ia | *G6PC1* | c.208 del T | Pathogenic | NA | NA | NA |  |
| p 07 | 1y/M | NO | Ib | *G6PT* | c.139_148+5delinsCA, C.945_964 | Pathogenic | NA | NA | NA |  |
| P 08 | 14y/M | YES | Ib | *G6PT* | c.1287_1290del | Pathogenic | NA | NA | NA |  |
|  |  |  |  |  |  |  |  |  |  |  |
| P 09 | 4m/M | NO | Ib | *G6PT* | p.D47H | VUS | 0.011 | -2.11 | 0.97 |  |
| P 10 | 8y/M | YES | Ib | *G6PT* | p.R300C | Likely pathogenic | -4.21 | 0 | 1 |  |
| P 11 | 5y/ M | YES | III | *AGL* | p.L35X | Pathogenic | NA | NA | NA |  |
| P 12 | 3y/F | YES | III | *AGL* | p.L35X | Pathogenic | NA | NA | NA |  |
| P 13 | 9y/ M | YES | III | *AGL* | p.T596X | Pathogenic | NA | NA | NA |  |
| P 14 | 3 y/ F | YES | III | *AGL* | p.Q833X | Pathogenic | NA | NA | NA |  |
| P 15 | 1y/F | YES | III | *AGL* | p.R910X | Pathogenic | NA | NA | NA |  |
| P 16 |  | YES | III | *AGL* | p.Q1072X | Pathogenic | NA | NA | NA |  |
| P 17 | 4 y/F | NO | III | *AGL* | p.Q1376X | Pathogenic | NA | NA | NA |  |
| P 18 | 1.6y/M | YES | III | *AGL* | p.Y1457X | Pathogenic | NA | NA | NA |  |
| P 19 | 2.4 Y | YES | III | *AGL* | p.D251Efs*23 | Pathogenic | NA | NA | NA |  |
| p 20 | 3.5y/M | NO | III | *AGL* | p.Leu316Profs*6 | Pathogenic | NA | NA | NA |  |
| P 21 | 7m/F | NO | III | *AGL* | p.Pro999Hisfs*13 | Pathogenic | NA | NA | NA |  |
| P 22 | 2y/M | YES | III | *AGL* | EXON 30-31del | Pathogenic | NA | NA | NA |  |
| P 23 | 4y/F | YES | III | *AGL* | p.Asn1304Lysfs*7 | Pathogenic | NA | NA | NA |  |
| P 24 | 4y/F | NO | III | *AGL* | p.L35X, c.1735+1G>T | Pathogenic/ Likely pathogenic | NA | NA | NA |  |
| P 25 | 8 y/ F | YES | III | *AGL* | c.664+1 G>C | Likely pathogenic | NA | NA | NA |  |
| P 26 | 3 y | YES | III | *AGL* | c.1423+1 G>C | Pathogenic | NA | NA | NA |  |
| P 27 | 3y/F | YES | III | *AGL* | c.1423+1G>A | Pathogenic | NA | NA | NA |  |
| P 28 | 2 y/ M | YES | III | *AGL* | c.1612-1G>A, p.D627G | Likely pathogenic | NA | NA | NA |  |
| P 29 | 3 y/M | YES | III | *AGL* | c.2681+1G>T | Pathogenic | NA | NA | NA |  |
| P 30 | 3 y/M | NO | III | *AGL* | c.2681+1G>T | Pathogenic | NA | NA | NA |  |
| P 31 | 3 y/ M | YES | III | *AGL* | c.2949+5G>A | Likely pathogenic | NA | NA | NA |  |
| P 32 | 6 y/ F | YES | III | *AGL* | c.2949+5G>A | Likely pathogenic | NA | NA | NA |  |
| P 33 | 10y/F | YES | III | *AGL* | c.3259+3A>T | Pathogenic | NA | NA | NA |  |
| p 34 | 2 y/ M | YES | III | *AGL* | p.H360Y, p.D627G | Pathogenic |  |  |  |  |
| P 35 | 2 y/ F | YES | III | *AGL* | p.H360Y, p.D627G | Pathogenic |  |  |  |  |
| P 36 | 9m/M | YES | III | *AGL* | p.H360Y, p.D627G | Pathogenic |  |  |  |  |
| P 37 | 6y/M | YES | III | *AGL* | p.R1121K | Likely pathogenic | -0.7 | 0.14 | 0.58 |  |
| P 38 | 1.2 y/ M | YES | III | *AGL* | p.X1533Y | Likely pathogenic | NA | NA | NA |  |
| P 39 | 8 y/ M | YES | VI | *PYGL* | c.1620+1G>C | Pathogenic | NA | NA | NA |  |
| P 40 | 4y/M | YES | VI | *PYGL* | c.1620+1G>C | Pathogenic | NA | NA | NA |  |
| P 41 | 5y/F | NO | VI | *PYGL* | c.1620+1G>C, p.N24K | Pathogenic/Pathogenic | NA | NA | NA |  |
| P 42 | 5y/M | YES | VI | *PYGL* | c.1620+1G>C | Pathogenic | NA | NA | NA |  |
| P 43 | 13y/M | YES | VI | *PYGL* | c.1620+1G>C | Pathogenic | NA | NA | NA |  |
| P 44 | 12y/F | YES | VI | *PYGL* | p.Arg12Alafs*99 | Pathogenic | NA | NA | NA |  |
| P 45 | 4y/F | N | VI | *PYGL* | p.G673K | Pathogenic | 0.001 | -3.79 | 0.98 |  |
| P 46 | 4y/M | NO | VI | *PYGL* | p.G686R | VUS | -7.22 | 0 | 1 |  |
| P 47 | 2y/M | NO | IXa | *PHKA2* | p.R45Q | Pathogenic | -3.61 | 0 | 0.999 |  |
| P 48 | 8m/M | NO | IXa | *PHKA2* | p.H957R | VUS | -6.97 | 0.005 | 0.968 |  |
| P 49 | 21y/M | YES | IXb | *PHKB* | c.(76+1_77-1)_(1068+1_1069-1)del | Pathogenic | NA | NA | NA |  |
| P 50 | 2Y/M | YES | IXb | *PHKB* | c(c.? _-1)_(c.1068+1_1069-1) del | Pathogenic | NA | NA | NA |  |
| P 51 | 6y/F | YES | IXb | *PHKB* | c.1364-2A>G | Pathogenic | NA | NA | NA |  |
| P 52 | 3y/ M | YES | IXc | *PHKG2* | p.V106E | Pathogenic | -5.56 | 0 | 0.999 |  |
| P 53 | 2.10 y/ F | YES | IXc | *PHKG2* | p.V106E | Pathogenic | -5.56 | 0 | 0.999 |  |
| P 54 | 7y/M | YES | IXc | *PHKG2* | p.V106E | Pathogenic | -5.56 | 0 | 0.999 |  |
| P 55 | 1.5Y/F | YES | IXc | *PHKG2* | p.E77K | Likely pathogenic | -3.79 | 0.001 | 0.997 |  |
| P 56 | 3y/M | YES | IXc | *PHKG2* | p.D215N | Likely pathogenic | -4.7 | 0 | 1 |  |
| P 57 | 19y/F | YES | IXc | *PHKG2* | p.Pro180LeufsTer15 | pathogenic | NA | NA | NA |  |

Table 8: Variations Identified in our study pathogenic, novel and VUS- variant of unknown significance(clinvar)

**Table 9:** Clinical profiling and molecular variants in 28 GSDIII affected individuals.

Variations and genotype- phenotype details of the GSDIII individuals. * - Novel variations, NC- not consanguineous, NA-not available.

| **Name** | **Gene(s)** | **Condition(s)** | **Clinical significance (Last reviewed)** |
| --- | --- | --- | --- |
| NM_000151.4(G6PC1):c.208del (p.Trp70fs) | G6PC1 | Glycogen storage disease due to glucose-6-phosphatase deficiency type IA | Pathogenic(Last reviewed: Jul 21, 2016) |
| NM_000151.4(G6PC1):c.355C>G (p.His119Asp) | G6PC1 | Glycogen storage disease due to glucose-6-phosphatase deficiency type IA | Pathogenic(Last reviewed: Sep 1, 2015) |
| NM_000151.4(G6PC1):c.468G>A (p.Trp156Ter) | G6PC1 | Glycogen storage disease due to glucose-6-phosphatase deficiency type IA | Pathogenic(Last reviewed: Jun 11, 2013) |
| NM_000151.4(G6PC1):c.550G>T (p.Gly184Ter) | G6PC1 | Glycogen storage disease due to glucose-6-phosphatase deficiency type IA | Pathogenic(Last reviewed: Aug 20, 2013) |
| NM_000151.4(G6PC1):c.664G>A (p.Gly222Arg) | G6PC1 | Glycogen storage disease due to glucose-6-phosphatase deficiency type IA | Pathogenic(Last reviewed: Aug 20, 2020) |
| NM_001164277.2(SLC37A4):c.1287_1290del (p.Ter430GluextTer?) | SLC37A4 | Glucose-6-phosphate transport defect | Conflicting interpretations of pathogenicity(Last reviewed: Jul 22, 2019) |
| NM_001164277.2(SLC37A4):c.945_964del (p.Met315fs) | SLC37A4 | Glucose-6-phosphate transport defect | Pathogenic(Last reviewed: Sep 20, 2020) |
| NM_001164277.2(SLC37A4):c.898C>T (p.Arg300Cys) | SLC37A4 | not provided\|Glucose-6-phosphate transport defect | Conflicting interpretations of pathogenicity(Last reviewed: Nov 15, 2021) |
| NM_001164277.2(SLC37A4):c.152TCA[1] (p.Ile52del) | SLC37A4 | Glucose-6-phosphate transport defect | Uncertain significance(Last reviewed: Jan 9, 2014) |
| NM_001164277.2(SLC37A4):c.139G>C (p.Asp47His) | SLC37A4 | Glucose-6-phosphate transport defect | Uncertain significance(Last reviewed: Sep 2, 2020) |
| NM_000642.3(AGL):c.104T>G (p.Leu35Ter) | AGL | Glycogen storage disease type III | Pathogenic/Likely pathogenic(Last reviewed: Jul 15, 2021) |
| NM_000642.3(AGL):c.664+1G>C | AGL | Glycogen storage disease type III | Likely pathogenic(Last reviewed: Jul 9, 2015) |
| NM_000642.3(AGL):c.753_756del (p.Asp251fs) | AGL | Glycogen storage disease type III\|not provided | Pathogenic(Last reviewed: Mar 22, 2022) |
| NM_000642.3(AGL):c.947_948del (p.Leu316fs) | AGL | Glycogen storage disease type III | Pathogenic(Last reviewed: Nov 8, 2016) |
| NM_000642.3(AGL):c.1078C>T (p.His360Tyr) | AGL | Glycogen storage disease type III\|not provided | Conflicting interpretations of pathogenicity(Last reviewed: Jul 15, 2021) |
| NM_000642.3(AGL):c.1423+1G>C | AGL | Glycogen storage disease type III | Pathogenic(Last reviewed: Jul 15, 2021) |
| NM_000642.3(AGL):c.1612-1G>A | AGL | Glycogen storage disease type III | Likely pathogenic(Last reviewed: Jun 15, 2020) |
| NM_000642.3(AGL):c.1735+1G>T | AGL | Glycogen storage disease IIIa\|Glycogen storage disease type III | Pathogenic/Likely pathogenic(Last reviewed: Nov 23, 2021) |
| NM_000642.3(AGL):c.1788T>G (p.Tyr596Ter) | AGL | Glycogen storage disease type III | Pathogenic(Last reviewed: Nov 3, 2014) |
| NM_000642.3(AGL):c.1880A>G (p.Asp627Gly) | AGL | Glycogen storage disease type III | Benign(Last reviewed: Jan 9, 2020) |
| NM_000642.3(AGL):c.2497C>T (p.Gln833Ter) | AGL | Glycogen storage disease type III | Pathogenic(Last reviewed: Apr 17, 2017) |
| NM_000642.3(AGL):c.2681+1G>T | AGL | Glycogen storage disease type III | Pathogenic(Last reviewed: Feb 8, 2022) |
| NM_000642.3(AGL):c.2728C>T (p.Arg910Ter) | AGL | Glycogen storage disease type III | Pathogenic(Last reviewed: Jul 15, 2021) |
| NM_000642.3(AGL):c.2949+5G>A | AGL | Glycogen storage disease type III | Likely pathogenic(Last reviewed: Nov 27, 2018) |
| NM_000642.3(AGL):c.2996del (p.Pro999fs) | AGL | Glycogen storage disease type III | Pathogenic(Last reviewed: Oct 9, 2020) |
| NM_000642.3(AGL):c.3214G>T (p.Glu1072Ter) | AGL | Glycogen storage disease type III | Pathogenic(Last reviewed: Aug 3, 2020) |
| NM_000642.3(AGL):c.3362G>A (p.Arg1121Lys) | AGL | Glycogen storage disease type III | Likely pathogenic(Last reviewed: Aug 27, 2020) |
| NM_000642.3(AGL):c.3911dup (p.Asn1304fs) | AGL | Glycogen storage disease type III | Pathogenic/Likely pathogenic(Last reviewed: Jul 15, 2021) |
| NM_000642.3(AGL):c.4126C>T (p.Gln1376Ter) | AGL | Glycogen storage disease type III | Pathogenic/Likely pathogenic(Last reviewed: Aug 4, 2019) |
| NM_000642.3(AGL):c.4371T>G (p.Tyr1457Ter) | AGL | Glycogen storage disease type III | Pathogenic(Last reviewed: Jun 1, 2017) |
| NM_002863.5(PYGL):c.2056G>C (p.Gly686Arg) | PYGL | Glycogen storage disease, type VI | Uncertain significance(Last reviewed: Aug 27, 2020) |
| NM_002863.5(PYGL):c.2017G>A (p.Glu673Lys) | PYGL | Glycogen storage disease, type VI | Uncertain significance(Last reviewed: Aug 14, 2021) |
| NM_002863.5(PYGL):c.1620+1G>C | PYGL | Glycogen storage disease, type VI | Pathogenic(Last reviewed: Aug 27, 2020) |
| NM_002863.5(PYGL):c.72C>A (p.Asn24Lys) | PYGL | Glycogen storage disease, type VI | Pathogenic(Last reviewed: Aug 27, 2020) |
| NM_002863.5(PYGL):c.33dup (p.Arg12fs) | PYGL | Glycogen storage disease, type VI | Pathogenic(Last reviewed: Aug 27, 2020) |
| NM_000293.3(PHKB):c.1364-2A>G | PHKB | Glycogen storage disease IXb | Pathogenic(Last reviewed: Aug 27, 2020) |
| NM_000293.3:c.(?_-1)_(1068+1_1069-1)del | PHKB | Glycogen storage disease IXb | Pathogenic(Last reviewed: Aug 27, 2020) |
| NM_000293.3:c.(76+1_77-1)_(1068+1_1069-1)del | PHKB | Glycogen storage disease IXb | Pathogenic(Last reviewed: Aug 27, 2020) |
| NM_000294.3(PHKG2):c.229G>A (p.Glu77Lys) | PHKG2 | Glycogen storage disease IXc | Pathogenic/Likely pathogenic(Last reviewed: Aug 27, 2020) |
| NM_000294.3(PHKG2):c.317T>A (p.Val106Glu) | PHKG2 | Glycogen storage disease IXc | Pathogenic(Last reviewed: Aug 27, 2020) |
| NM_000294.3(PHKG2):c.539del (p.Pro180fs) | PHKG2 | Glycogen storage disease IXc | Pathogenic(Last reviewed: Aug 27, 2020) |
| NM_000294.3(PHKG2):c.643G>A (p.Asp215Asn) | PHKG2 | Glycogen storage disease IXc\|not specified | Conflicting interpretations of pathogenicity(Last reviewed: Jul 22, 2021) |

Table 10: HGVS Nomenclature with Clinical significance.

**Comparative analysis of amino acid sequences**

H119D G222R Lys76Arg

[NP_000142.2](https://www.ncbi.nlm.nih.gov/entrez/viewer.fcgi?db=protein&id=393537031) (human)   PGSPSGHAMGT FSFAIGFYL  NLVFKWILF

[XP_003315515.1](https://www.ncbi.nlm.nih.gov/entrez/viewer.fcgi?db=protein&id=332847759) (chimpanzee) PGSPSGHAMGT FSFAIGFYL NLVFKWILF

[XP_001112333.1](https://www.ncbi.nlm.nih.gov/entrez/viewer.fcgi?db=protein&id=109115541) (monkey) PGSPSGHAMGT FSFAIGFYL NLVFKWILF

NP_001002993.1 (dog) PGSPSGHAMGT FSFAIGFYL NLVFKWILF

[NP_001069592.1](https://www.ncbi.nlm.nih.gov/entrez/viewer.fcgi?db=protein&id=115497704) (cow) PGSPSGHAMGT FSFAIGFYL NLVFKWILF

[NP_032087.2](https://www.ncbi.nlm.nih.gov/entrez/viewer.fcgi?db=protein&id=31982353) (mouse     PGSPSGHAMGA FGFALGFYL NLVFKWILF

[NP_037230.2](https://www.ncbi.nlm.nih.gov/entrez/viewer.fcgi?db=protein&id=145207998) (rat)      PGSPSGHAMGT FGFALGFYL NLVFKWILF

[XP_003642865.1](https://www.ncbi.nlm.nih.gov/entrez/viewer.fcgi?db=protein&id=363743532)(chiken) PGSPSGHAMGA FSFALGFYL NLVFKWILF

[NP_001157278.1](https://www.ncbi.nlm.nih.gov/entrez/viewer.fcgi?db=protein&id=255069754)(zebra fish) PGSPSGHAMGA FSFAVGLYV NLVFKWILF

[NP_001096531.1](https://www.ncbi.nlm.nih.gov/entrez/viewer.fcgi?db=protein&id=156717978) (clawed frog) PGSPSGHAMGSA FSFALGFYL NLVFKWILF

**Figure 1 : GSD1a**

D47H R300C p.Glu429ext*52

[NP_001157750.1](https://www.ncbi.nlm.nih.gov/entrez/viewer.fcgi?db=protein&id=256219543)(human) PLDKDDLGF GNPRHGLL MGRVSKKAE

[XP_508803.2](https://www.ncbi.nlm.nih.gov/entrez/viewer.fcgi?db=protein&id=114640692) (chimpanzee)     PLDKDDLGF GNPRHGLL MGRVSKKAE

[XP_001100471.1](https://www.ncbi.nlm.nih.gov/entrez/viewer.fcgi?db=protein&id=109108894)( rhesus macaque) PLDKDDLGL GNPRHGLL MGRVSKKAE

[XP_546493.2](https://www.ncbi.nlm.nih.gov/entrez/viewer.fcgi?db=protein&id=73954666) (dog)     PLDKDDLGL GNPRHGLL MGRVPKKAE

[NP_001192279.1](https://www.ncbi.nlm.nih.gov/entrez/viewer.fcgi?db=protein&id=329112559) (cow) PLDKDDLGL GNPRHGLL MGRVPKKAE

[NP_032089.2](https://www.ncbi.nlm.nih.gov/entrez/viewer.fcgi?db=protein&id=51592076) (mouse)     ALDKDDLGL GNPRHGLL MGRVSKKAE

[NP_113777.2](https://www.ncbi.nlm.nih.gov/entrez/viewer.fcgi?db=protein&id=47575861)(rat)     ALDKDDLGL GNPRHSLL MGRVSKKAE

[XP_001233002.1](https://www.ncbi.nlm.nih.gov/entrez/viewer.fcgi?db=protein&id=118102039)(chicken) PLDKDDLGL GNPRHTLL MGRVSKKAE

[XP_005169262.1](https://www.ncbi.nlm.nih.gov/entrez/viewer.fcgi?db=protein&id=528492859)(zebra fish) ELDKEELGL GNPRHGLL MGHIPRKAD

[NP_001017356.1](https://www.ncbi.nlm.nih.gov/entrez/viewer.fcgi?db=protein&id=62860104) (clawed frog) ELDKEELGL GNPRHGLL MGHIPRKAD

**Figure 2: GSD1b**

**Figure 3: GSDIII**

N24K E673K G686R

[NP_002854.3](https://www.ncbi.nlm.nih.gov/entrez/viewer.fcgi?db=protein&id=71037379) (Human)     VGVENVAELK AGTEASGTG FMLNGALTI

[XP_001145433.2](https://www.ncbi.nlm.nih.gov/entrez/viewer.fcgi?db=protein&id=332842210) (Chimpanzee) VGVENVAELK AGTEASGTG FMLNGALTI

[XP_001102253.1](https://www.ncbi.nlm.nih.gov/entrez/viewer.fcgi?db=protein&id=109083559) (Rhesus) VGVENVAELK AGTEASGTG FMLNGALTI

[XP_003639242.1](https://www.ncbi.nlm.nih.gov/entrez/viewer.fcgi?db=protein&id=359320053) (wolf) VGVENVAELK AGTEASGTG FMLNGALTI

[NP_001068671.1](https://www.ncbi.nlm.nih.gov/entrez/viewer.fcgi?db=protein&id=115498012) (Cow) VGVENVAELK AGTEASGTG FMLNGALTI

[NP_573461.2](https://www.ncbi.nlm.nih.gov/entrez/viewer.fcgi?db=protein&id=268836255) (House mouse)  VGVENVAELK AGTEASGTG FMLNGALTI

[NP_071604.1](https://www.ncbi.nlm.nih.gov/entrez/viewer.fcgi?db=protein&id=11560087) (Brown Rat)    VGVENVAELK AGTEASGTG FMLNGALTI

[NP_989723.1](https://www.ncbi.nlm.nih.gov/entrez/viewer.fcgi?db=protein&id=45383372) (Fowl)     VGAENVAELK AGTEASGTG FMLNGALTI

[XP_002936619.1](https://www.ncbi.nlm.nih.gov/entrez/viewer.fcgi?db=protein&id=301614311) (frog) VGVENVAELK AGTEASGTG FMLNGALTI

**Figure 4 : GSDVI**

R45Q H957R

[NP_000283.1](https://www.ncbi.nlm.nih.gov/entrez/viewer.fcgi?db=protein&id=4505781) (Human)     AWVRDNIY NLLHHILSG

[XP_003317431.1](https://www.ncbi.nlm.nih.gov/entrez/viewer.fcgi?db=protein&id=332860412) (Chimpanzee) AWVRDNIY NLLHHILSG

[XP_001084454.1](https://www.ncbi.nlm.nih.gov/entrez/viewer.fcgi?db=protein&id=109130112) (Rhesus) AWVRDNIY NLLHHILSG

[XP_005641224.1](https://www.ncbi.nlm.nih.gov/entrez/viewer.fcgi?db=protein&id=545557607) (wolf) AWVRDNIY NLLHHILSG

[NP_001178474.1](https://www.ncbi.nlm.nih.gov/entrez/viewer.fcgi?db=protein&id=329112491) (Cow) AWVRDNIY NLLHHILSG

[NP_001171350.1](https://www.ncbi.nlm.nih.gov/entrez/viewer.fcgi?db=protein&id=295424154) (House mouse) AWVRDNIY SLLHHILSG

[NP_001177923.1](https://www.ncbi.nlm.nih.gov/entrez/viewer.fcgi?db=protein&id=300253230) (Brown Rat) AWVRDNIY SLLHHILSG

[XP_004934744.1](https://www.ncbi.nlm.nih.gov/entrez/viewer.fcgi?db=protein&id=513163549) (Fowl) AWVRDNVY NLLHHILSG

[XP_684044.4](https://www.ncbi.nlm.nih.gov/entrez/viewer.fcgi?db=protein&id=528494636) (frog)   AWVRDNVY NLLHHILSG

[NP_001138180.1](https://www.ncbi.nlm.nih.gov/entrez/viewer.fcgi?db=protein&id=221329791) AWIRDNVY NLLHHILSG

**Figure 5: GSDIXa**

E77K V106E D215N

| [NP_000285.1](https://www.ncbi.nlm.nih.gov/entrez/query.fcgi?cmd=Retrieve&db=protein&list_uids=4505785&dopt=GenPept&term=4505785&qty=1&linkbar=jsmenu2) (Human) RLSPEQLEE SFMFEVLVFD GKEVDLWACG |
| --- |
| [XP_001144282.2](https://www.ncbi.nlm.nih.gov/entrez/query.fcgi?cmd=Retrieve&db=protein&list_uids=332845736&dopt=GenPept&term=332845736&qty=1&linkbar=jsmenu2) (Chimpanzee) RLSPEQLEE SFMFEVLVFD GKEVDLWACG |
| [XP_002802499.1](https://www.ncbi.nlm.nih.gov/entrez/query.fcgi?cmd=Retrieve&db=protein&list_uids=297283845&dopt=GenPept&term=297283845&qty=1&linkbar=jsmenu2) (Rhesus) RLSPEQLEE SFMFEVLVFD GKEVDLWACG |
| [XP_005621329.1](https://www.ncbi.nlm.nih.gov/entrez/query.fcgi?cmd=Retrieve&db=protein&list_uids=545501465&dopt=GenPept&term=545501465&qty=1&linkbar=jsmenu2) (wolf) RLSPEQLEE SFMFEVLVFD SKEVDLWACG |
| [NP_001039593.1](https://www.ncbi.nlm.nih.gov/entrez/query.fcgi?cmd=Retrieve&db=protein&list_uids=114050953&dopt=GenPept&term=114050953&qty=1&linkbar=jsmenu2) (Cow) RLSPEQLEE SFMFEVLVFD GKEVDLWACG |
| [NP_081164.2](https://www.ncbi.nlm.nih.gov/entrez/query.fcgi?cmd=Retrieve&db=protein&list_uids=188035881&dopt=GenPept&term=188035881&qty=1&linkbar=jsmenu2) (House mouse) RLSPEQLEE SFMFEVLVFD GKEVDLWACG |
| [NP_957256.1](https://www.ncbi.nlm.nih.gov/entrez/query.fcgi?cmd=Retrieve&db=protein&list_uids=41055716&dopt=GenPept&term=41055716&qty=1&linkbar=jsmenu2) (Zebra fish) RLSPEQLEE SFMFEVLVFD GKEVDLWACG |
| [NP_727548.1](https://www.ncbi.nlm.nih.gov/entrez/query.fcgi?cmd=Retrieve&db=protein&list_uids=24641361&dopt=GenPept&term=24641361&qty=1&linkbar=jsmenu2) (Drosophila) KMTEQQLEE AFIFLVFD SQEVDIWACG |
| [XP_312280.3](https://www.ncbi.nlm.nih.gov/entrez/query.fcgi?cmd=Retrieve&db=protein&list_uids=158290709&dopt=GenPept&term=158290709&qty=1&linkbar=jsmenu2) (Mosquito) ESGETNPYH AFVFLVFD SKEVDIWACG  **Figure 6: GSDIXc** |
|  |
